# A family-wide assessment of latent STAT transcription factor interactions reveals divergent dimer repertoires

**DOI:** 10.1016/j.jbc.2023.104703

**Published:** 2023-04-12

**Authors:** Andreas Begitt, Sebastian Krause, James R. Cavey, Doratha E. Vinkemeier, Uwe Vinkemeier

**Affiliations:** 1 School of Life Sciences, The University of Nottingham, Nottingham, UK; 2 School of Computer Science, Nottingham Trent University, Nottingham, UK

**Keywords:** STAT1, STAT2, STAT3, STAT4, STAT5A, STAT5B, STAT6, transcription factor, dimerization, homodimer, heterodimer, interaction assay, nuclear export signal, nuclear import signal

## Abstract

The conversion of signal transducer and activator of transcription (STAT) proteins from latent to active transcription factors is central to cytokine signaling. Triggered by their signal-induced tyrosine phosphorylation, it is the assembly of a range of cytokine-specific STAT homo- and heterodimers that marks a key step in the transition of hitherto latent proteins to transcription activators. In contrast, the constitutive self-assembly of latent STATs and how it relates to the functioning of activated STATs is understood less well. To provide a more complete picture, we developed a co-localization-based assay and tested all 28 possible combinations of the seven unphosphorylated STAT (U-STAT) proteins in living cells. We identified five U-STAT homodimers—STAT1, STAT3, STAT4, STAT5A, and STAT5B—and two heterodimers—STAT1:STAT2 and STAT5A:STAT5B—and performed semi-quantitative assessments of the forces and characterizations of binding interfaces that support them. One STAT protein—STAT6—was found to be monomeric. This comprehensive analysis of latent STAT self-assembly lays bare considerable structural and functional diversity in the ways that link STAT dimerization before and after activation.

The signal transducer and activator of transcription (STAT) proteins are an evolutionarily conserved family of seven transcription factors in mammals, namely, STAT1, STAT2, STAT3, STAT4, STAT5A, STAT5B, and STAT6 (1). These proteins are the substrate of both receptor and non-receptor tyrosine kinases such as the Janus kinases (JAK), which catalyze their phosphorylation of a single C-terminal tyrosine residue after ligand binding to cell surface receptors (2). About 50 different growth factors and cytokines including interferons, interleukins, and growth hormones are known to signal *via* STAT proteins (3). The activated STATs accumulate in the nucleus and participate in the transcription of hundreds of genes (4). This sequence of events, usually referred to as canonical JAK-STAT signaling, entails the dimerization of STAT proteins through mutual phosphotyrosine:SH2 domain interactions. Such dimers can bind short palindromic stretches of DNA called GAS elements in the promoter region of target genes (5). In addition to homodimers, activated STATs can assemble heterodimers with the other family members (6).

While the phosphotyrosine-mediated functions of STATs were linked to their dimerization early on (7), much less is known to date regarding the constitutive self-assembly of latent, that is, unphosphorylated STATs (U-STATs). Initially, U-STATs were believed to be monomeric (7), but further studies showed that STATs can assemble high-molecular-weight complexes already before tyrosine phosphorylation (8, 9). A well-documented example is STAT1, which forms equally strong dimers before and after activation as shown by analytical ultracentrifugation (10). Importantly, crystallographic evidence demonstrates that unphosphorylated dimers of STAT1, STAT3, and STAT5 are stabilized not by interactions of the carboxy-terminal SH2 domains but by interactions between amino-terminal regions (11, 12, 13), resulting in antiparallel orientation of monomers as opposed to their parallel orientation in the phosphodimers (14). It has been recognized that unphosphorylated STATs are involved in many biological events in both normal and pathological situations (15, 16, 17, 18, 19). In some instances, the self-assembly of U-STATs has been linked to tyrosine phosphorylation and cytokine signaling. For example, the homodimerization of U-STAT4 is a prerequisite for cytokine-induced STAT4 activation (20), and heterodimerization of U-STAT1 with U-STAT2 has been shown to have positive or negative consequences for type 1 and type 2 interferon signaling, respectively (21, 22). These and other findings indicate that U-STAT dimers fulfill roles critical for cytokine functioning, but the knowledge of dimerization before cytokine-induced phosphorylation remains incomplete. This is particularly true for heterotypic interactions across the STAT family and the *in vivo* situation generally. To fill in knowledge gaps, we devised an assay to systematically explore the repertoires of homo- and heterotypic interactions among U-STATs in living cells. The assay is based on U-STATs being nucleocytoplasmic shuttling proteins that can freely cross the soft diffusion barrier posed by the nuclear pore, presumably *via* direct contact with nuclear pore proteins (23, 24). It results in generally pancellular distributions of U-STATs (25), which for STAT1 and STAT2 can be shifted to nuclear or cytoplasmic accumulation by tagging them with transferable carrier-dependent nuclear localization (NLS) or nuclear export (NES) signals (26). We reasoned that such signal-tagged variants might function as baits that attract co-expressed test proteins if binding interactions occurred. This would be evident by their co-localization in the bait protein’s compartment. After rigorous testing and verification, this approach was used to probe homo- and heterotypic binding interactions within cells across the entire STAT family for the first time.

## Results

### Co-localization as an assay to detect the dimerization of U-STATs within cells

**Figure 1:**
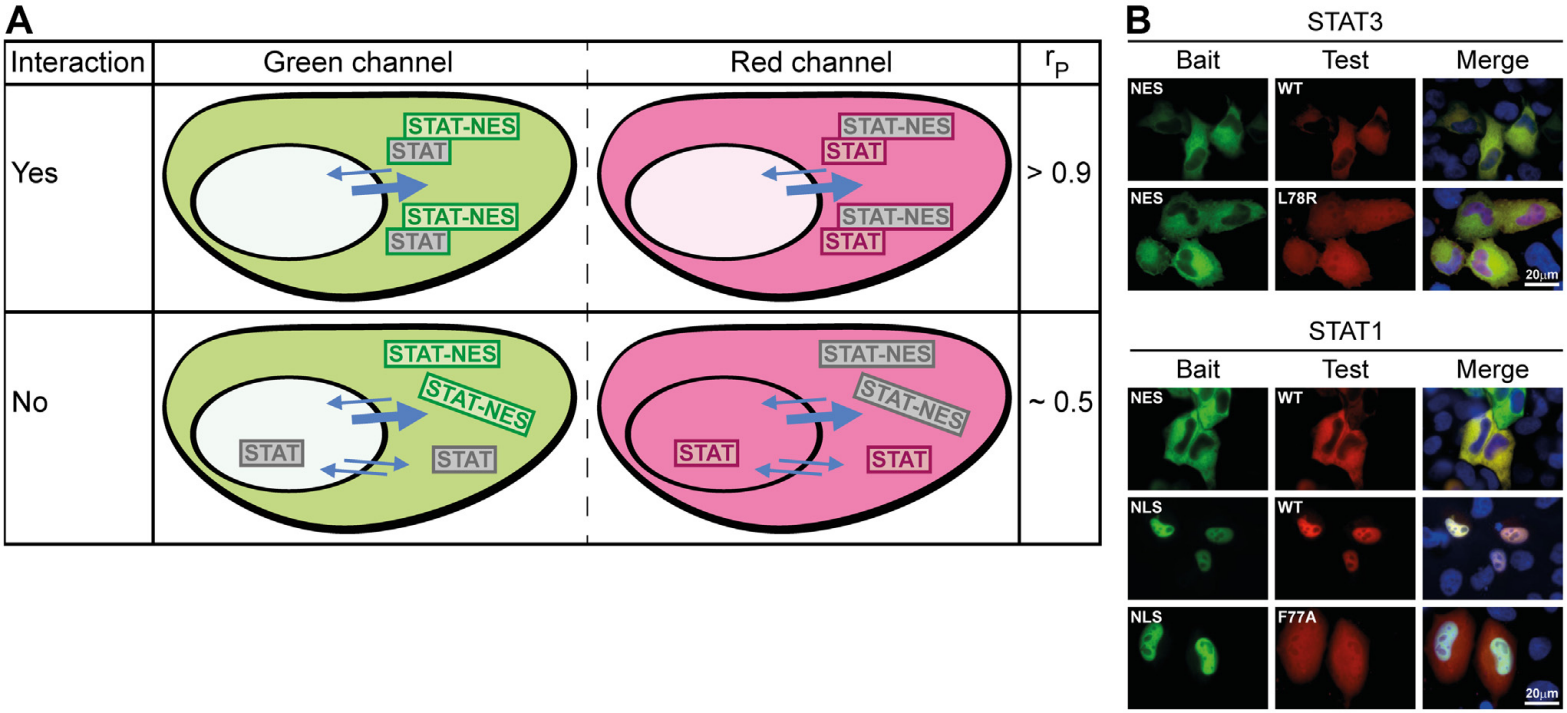
**Outline and validation of co-localization as an assay to probe dimerization of latent STATs.***A*, schematics describing expected subcellular distributions of (*top*) interacting and (*bottom*) non-interacting bait (*green fluorescence*) and test proteins (*red fluorescence*). Shown are the anticipated outcomes obtained in *green* and *red channels* together with associated Pearson correlation coefficients, r_P_, which quantify the degree of co-localization of the fluorophores, obtained with the co-localization assay. The bait protein is fused to a nuclear export signal (NES), directing it to the cytoplasm, whereas the test protein is without heterologous translocation signal. *Arrow* orientation and width signify the direction and relative efficiency of nucleocytoplasmic translocation of the different protein species. *B*, fluorescence micrographs of representative HeLa cells co-expressing bait and test protein combinations for STAT3 (*top*) and STAT1 (*bottom*). Baits were directed to the cytoplasm or nucleus through appended NES or NLS signal sequences as indicated in the panels. Homotypic STAT protein interactions were probed by co-expressing bait and wild-type (WT) STATs; mutant STAT1 (F77A) and STAT3 (L78R) were used to examine the consequences of known dimer-dissociating mutations on the co-localization of bait and test STAT proteins. Shown are *green channel*, *red channel*, and merged channels, which includes visualization of nuclei using Hoechst dye (*blue*).

**Supporting Figure S1:**
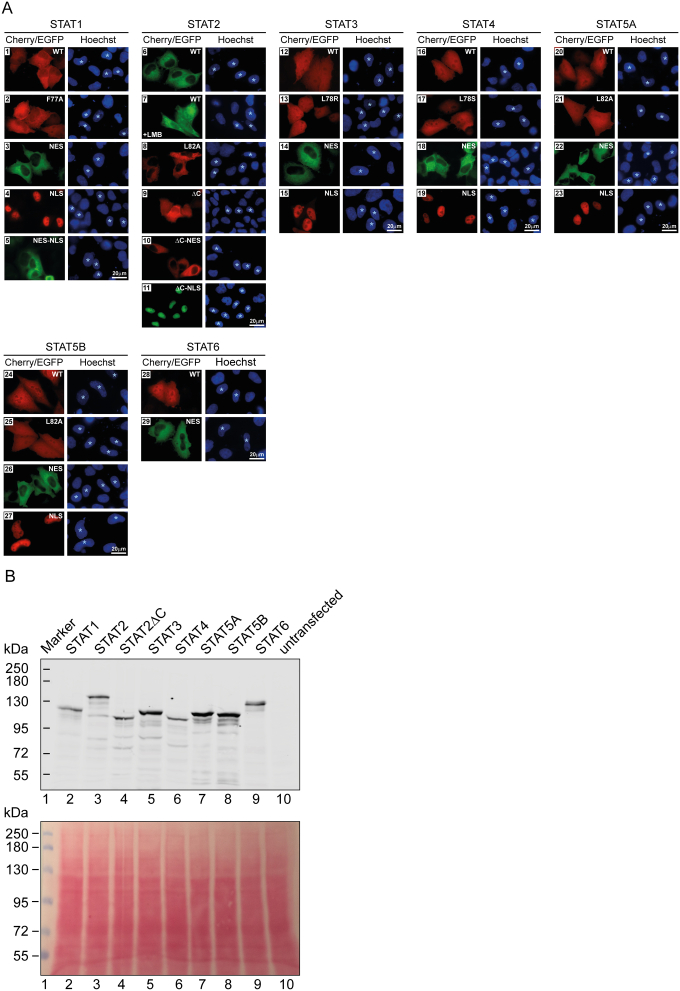
**Characterization of STAT variant proteins used.***A*, Representative micrographs of HeLa cells expressing the indicated STAT variant proteins. The intracellular distributions of the following STAT-fluorophore fusion proteins are shown. Wild-type STAT1-6; N-domain mutated STATs (F77A, L78R/S, L82A); full-length STATs with heterologous signal sequence for either nuclear export (NES) or nuclear import (NLS) appended; full-length STAT1 with both the export and import signal sequences appended (NES-NLS); truncated STAT2 devoid of the C-terminus (ΔC); C-terminally truncated STAT2 with heterologous signal sequence for either nuclear export (NES) or nuclear import (NLS) appended. Presented are the *green* or *red channels*, and the Hoechst-stained cell nuclei (*blue*). Where there was treatment with leptomycin B (+LMB) it was for 4 h. The nuclei of interest are labelled with white *asterisks* to facilitate the matching of the GFP/Cherry signals and the Hoechst staining. *B*, Western blotting results using whole cell lysates (30 μl/lane) from HEK293T cells expressing mCherry fusion proteins of the full-length STATs or C-terminally truncated STAT2, as indicated. After blotting, total protein was stained with Ponceau S (*bottom panel*), followed by incubation with anti-mCherry antibody and immunodetection (*top panel*). In brief, experimental details were as follows. Twenty hours after transient transfection with vectors encoding STAT-mCherry fusion proteins as indicated, 4 × 10^6^ HEK293T cells (transfection efficiency ∼70%) were lysed by boiling in 0.5 ml 2× SDS sample buffer (42 mM Tris-HCl, pH 6.8, 1.3% SDS (w/v), 233 mM DTT, 8.3% glycerol (v/v), 0.04 % Bromphenol blue). Proteins (30 μl/lane) were resolved by 7% SDS-PAGE followed by semi-dry protein transfer to a nitrocellulose membrane. Total protein loading was visualized by staining with 0.1% (w/v) Ponceau S in 1% (v/v) acetic acid for 1 min. Excess stain was removed with water (1 min), followed by blocking in 4% (w/v) dried skimmed milk powder in Tris-buffered saline containing 0.05% (v/v) Tween 20 (Sigma) and incubation with rabbit polyclonal anti-mCherry antibody (GTX128509, GeneTex). IRDye 800CW-coupled goat anti-rabbit secondary antibody (#926-32211) was from Li-Cor Bioscience. Blots were imaged by infrared fluorescence detection (Odyssey, Li-Cor Bioscience).

Latent STATs are nucleocytoplasmic shuttling proteins that can freely cross the nuclear envelope by directly contacting nuclear pore proteins and additional carrier-mediated mechanisms (23). Accordingly, they display near pancellular distributions in cells before cytokine treatment (with the exception of STAT2, see below), which is preserved upon C-terminal fusion of fluorescent marker proteins such as mEGFP or mCherry (Fig. S1*A*, panels 1,12,16,20,24,28). A Western blot demonstrating the expression of full-length STAT-fluorophore fusion proteins is shown in Fig. S1*B*. As mentioned earlier, STAT2 deviates from the near-pancellular distribution of the other STAT proteins; it accumulates in the cytoplasm due to potent nuclear export activity in its C-terminal transactivation domain (Fig. S1*A*, panel 6). Consistent with previous observations (26, 27), STAT2 variants with truncated transactivation domain, referred to as STAT2ΔC, showed strongly reduced cytoplasmic accumulation, thus adopting a distribution more like the other STAT family members (Fig. S1*A*, panel 9). The addition of transferable heterologous nuclear export (NES) or nuclear import signals (NLS) to wild-type STATs (or the C-terminally truncated STAT2) allowed us to direct bait proteins to the cytoplasm or the nucleus, respectively (Fig. S1*A*, panels 3,4,10,11,14,15,18,19,22,23,26,27,29). We used two well-characterized and highly active signals, namely, an NES derived from protein kinase A inhibitor and the NLS from simian virus 40 large T-antigen (28, 29). These nuclear translocation-related features potentially allow STATs to alter the localization of proteins they interact with. Schematics of the assay design are shown in Figure 1*A*.

Another important aspect of the assay we considered was the strength of binding interactions, as this posed a main constraint on whether they are detectable as co-localization. Current data on U-STAT dimer assembly is largely qualitative; however, equilibrium sedimentation studies indicate that unphosphorylated STAT1 and STAT3 form high-affinity homodimers, both with a Kd in the low nanomolar range (10, 30). Moreover, structural and mutational studies of the dimer interfaces have identified the conserved N-domain and key anchoring residues therein as critical for unphosphorylated dimer assembly. Unphosphorylated STATs with N-domain truncation or mutated dimerization hot spot residues retain nucleocytoplasmic shuttling and their subcellular distributions differ little from their wild-type counterparts (31, 32) (see also Fig. S1*A*, panels 2,8,13,17,21,25). Nonetheless, these mutations render them essentially monomeric, as quantitative analyses of N-domain-deleted or residue F77-mutated STAT1 showed a >100-fold drop in binding affinities (10, 12). For STAT3, mutation of residue L78 similarly results in unphosphorylated dimer dissociation in living cells as demonstrated by Förster resonance energy transfer (31). These established results allowed us to use wild-type and mutant STAT1 and STAT3 as positive and negative controls to test if the co-localization of STAT proteins could be a reliable indicator for their dimerization inside cells. As shown in Figure 1*B*, co-expression of NES-fused STAT3 with wild-type STAT3 indeed resulted in their co-localization in the cytoplasm. Importantly, co-localization was lost if the monomeric STAT3-L78R mutant was used, which displayed pancellular distribution irrespective of the presence of STAT3-NES. This was the expected behavior of non-interacting, monomeric STAT3, in accord with the aforementioned *in vitro* and cellular studies. We then examined the homodimerization of STAT1 to validate further the co-localization assay. In the same way that STAT3 interacted with STAT3-NES, wild-type STAT1 co-localized with STAT1-NES in the cytoplasm, suggestive of homodimerization (Fig. 1*B*). To examine whether dimerization can also occur in the cell nucleus, we co-expressed STAT1 and nuclear-accumulated STAT1-NLS. As shown in Figure 1*B*, this likewise resulted in the co-localization of the two STAT1 variant proteins, albeit in the nucleus, demonstrating that dimerization was not limited to the cytoplasmic compartment. To test the consequences of a dimer-disrupting mutation for STAT1 homodimers, we used the mutant STAT1-F77A. In contrast to wild-type STAT1, the F77A mutant did not accumulate in the nucleus with co-expressed STAT1-NLS but retained pancellular distribution, similar to STAT3-L78R, thus again presenting the expected behavior of monomeric STATs. Finally, it was important to ascertain that the failure of a test protein to co-localize with bait was correctly attributed to a lack of dimer formation, rather than a shortfall of bait protein expression. Therefore, the relative expression of test and bait proteins was determined using quantitative fluorescence imaging as described in Experimental procedures, and cells were disregarded for inclusion in co-localization analyses if the expression of bait variants was below equimolar levels or exceeded the test protein’s concentration by more than 4-fold.

In summary, co-localization in nuclear or cytoplasmic compartments was a reliable indicator to assess the homodimerization of unphosphorylated STAT1 and STAT3. We, therefore, expanded this approach to assess the homodimer formation of all seven STATs as well as their ability to heterodimerize.

### Latent STATs assemble five homodimers and two heterodimers

**Figure 2:**
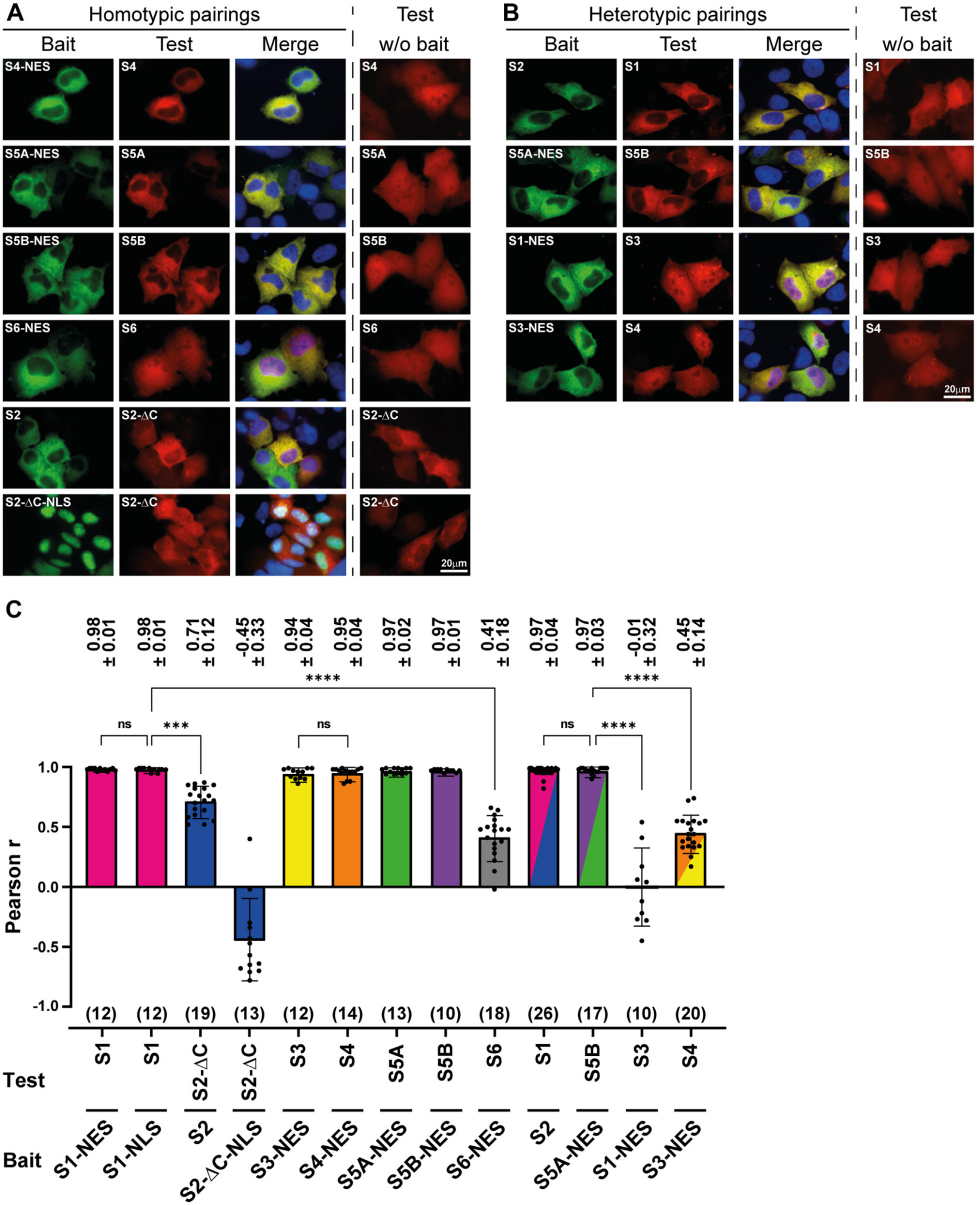
**Latent STATs assemble five homodimers and two heterodimers *via* N-domain interactions.***A* and *B*, fluorescence micrographs of representative HeLa cells co-expressing the indicated homotypic (*A*) and heterotypic (*B*) bait and test STAT protein pairings. Shown are the *green channel*, *red channel*, and merged channels, which includes visualisation of nuclei using Hoechst dye (*blue*). The rightmost columns show the distribution of test proteins in cells that do not co-express the bait (w/o bait). *C*, bar diagram depicting corresponding Pearson correlation coefficients (r_P_) for the experiments shown in (*A*) and (*B*) with the individual data points (*black dots*) super imposed. Co-localization was determined in eligible cells, that is, cells that expressed bait and test proteins from equimolar amounts up to a 4-fold excess of bait protein. ∗*p* < 0.05; ∗∗*p* < 0.01; ∗∗∗*p* < 0.001; ∗∗∗∗*p* < 0.0001 using a Kruskal–Wallis test; ns, not significant. Rp numerical values ± SD are given above the bars; the number of cells analyzed in each experiment are shown in brackets below bars. Error bars, SD.

**Figure 3:**
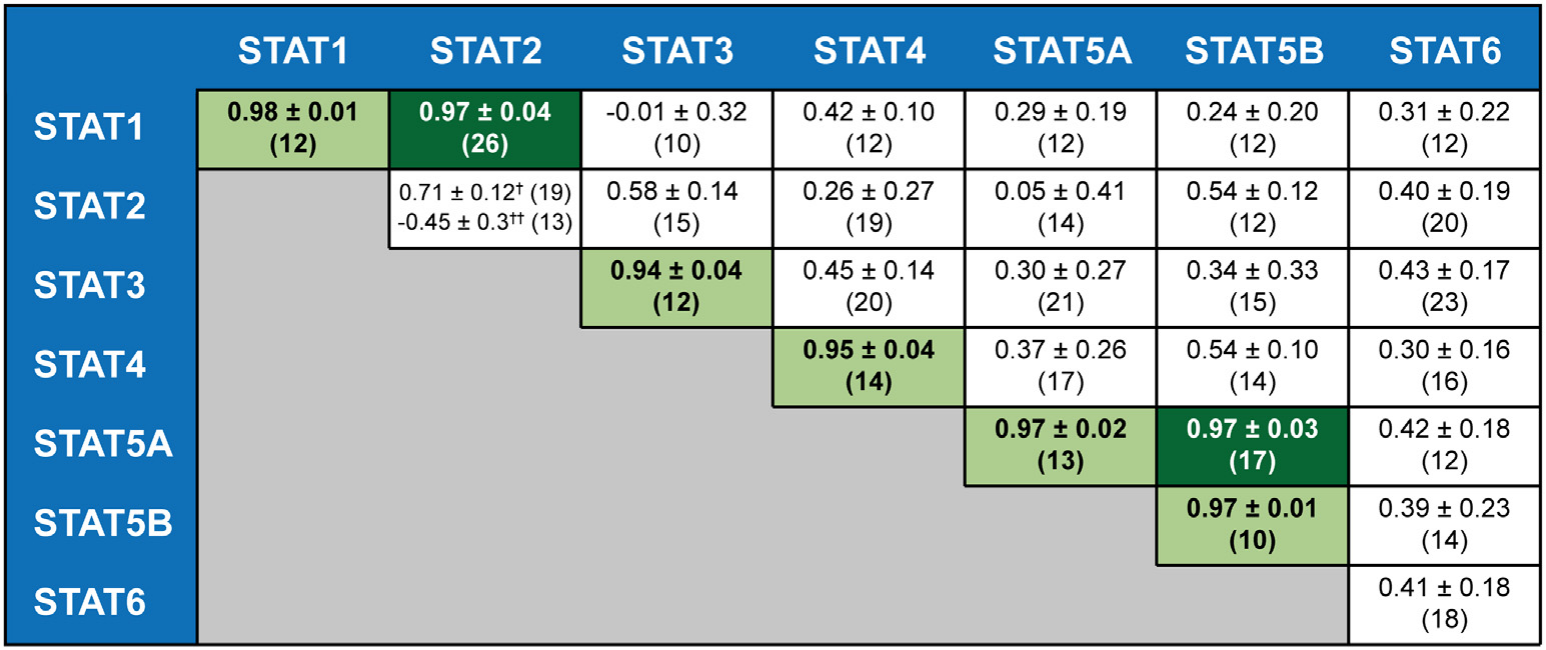
**Homo- and heterotypic dimerization of the unphosphorylated STAT proteins.** Summary of Pearson correlation coefficients obtained with the translocation assay for the 28 possible pairings of unphosphorylated STAT proteins. Data are obtained with NES fusion proteins and wild-type STAT2 as the baits. STAT2 homodimer data are for U-STAT2:STAT2ΔC (†) and U-STAT2ΔC-NLS:STAT2ΔC (††). Given are means ± standard deviation. *Light* and *dark green* highlighting marks stable homo- and heterodimers, respectively. Number of cells analyzed in each experiment are shown in *brackets*. See data availability section for source data.

**Supporting Figure S2:**
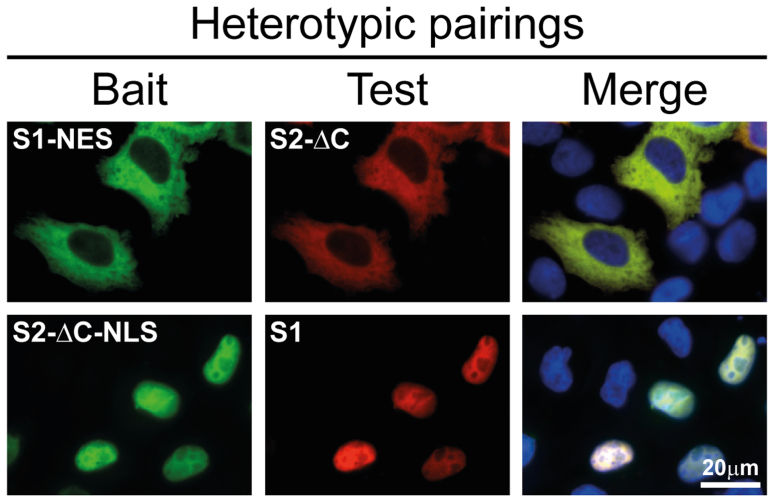
**U-STAT1:STAT2 heterodimers do not require the C-terminal transactivation domain of STAT2.** Representative micrographs of HeLa cells expressing the indicated bait and test protein pairings of variants of STAT1 and C-terminally truncated STAT2. (S1) wild-type STAT1; (S1-NES) full-length STAT1 with heterologous nuclear export signal appended; (S2-ΔC) truncated STAT2 devoid of the C-terminus and its intrinsic nuclear export activity; (S2-ΔC-NLS) C-terminally truncated STAT2 with heterologous nuclear import signal appended. Presented are the *green channel*, *red channel*, and merged channels, which includes the Hoechst-stained cell nuclei (*blue*).

To similarly examine the homodimerization of the other five STATs, we co-expressed STAT4, STAT5A, STAT5B, and STAT6 with their respective NES-tagged counterparts. As shown in Figure 2*A*, STAT6 failed to accumulate in the cytoplasm, in stark contrast to STAT4, STAT5A, and STAT5B, which co-localized with their NES-tagged equivalents. We quantified the extent of co-localization for each experiment by calculating Pearson’s correlation coefficients (*r*_P_) for 10 to 30 cells. Numerical values for homodimers are shown in Figure 2*C* and summarized in Figure 3. STAT1, STAT3, STAT4, STAT5A, and STAT5B have Pearson’s correlation coefficients of 0.94 or higher, indicative of near-complete co-localization due to stable homodimerization. STAT6 diverged strongly as it showed no cytoplasmic co-localization, with an accordingly significantly lower *r*_P_ value of 0.41. To assess the homodimerization of STAT2, the C-terminally truncated variant STAT2ΔC with strongly reduced intrinsic nuclear export activity was used, as described earlier (Fig. S1*A*, panel 9). Although the C-terminus is needed for efficient constitutive nuclear export of STAT2, this region generally appears to be dispensable for the dimerization of U-STATs (10, 33), including the heterodimerization of U-STAT2ΔC and U-STAT1, which was demonstrated using STAT1-NES and STAT2ΔC-NLS as baits (Fig. S2). The C-terminally truncated STAT2 mutant was therefore co-expressed with wild-type STAT2 to probe U-STAT2 homodimerization. We observed incomplete cytoplasmic co-localization (Fig. 2*A*) and a correlation coefficient *r*_P_ = 0.71 that was significantly reduced compared to stable dimers of other STAT family members yet higher than for non-interacting monomeric STAT6 (Fig. 2*C*). This could signify genuine homodimer assembly, albeit with lower affinity, or merely constitute an artifact reflecting co-localization due to residual cytoplasmic accumulation of STAT2ΔC. To distinguish between these possibilities, STAT2ΔC was directed to the nucleus through its tagging with an NLS (Fig. S1*A*, panel 11) to examine if this resulted in the nuclear localization of co-expressed STAT2ΔC. This was not the case, however, as the slight cytoplasmic accumulation of STAT2ΔC appeared unaltered, with no indication of increased nuclear translocation in the presence of the nuclear accumulated STAT2 bait protein. The correlation coefficient accordingly dropped sharply into the negative, *r*_P_ = −0.45 (Fig. 2*C*) which indicated opposite distributions and hence a lack of co-localization of the two STAT2 variants. We concluded that unphosphorylated STAT2, like STAT6, did not homodimerize, in contrast to STAT1, STAT3, STAT4, STAT5A, and STAT5B, which formed stable homodimers in living cells. Next, we probed the heterodimer assembly of the seven unphosphorylated STATs by co-expressing NES-tagged STAT proteins as baits (except STAT2, where wild-type was used) and untagged wild-type STATs as the test proteins. Of the 21 possible heterotypic pairings, only two showed co-localization of bait and test proteins, namely, U-STAT1:STAT2 and U-STAT5A:STAT5B (Fig. 2, *B* and *C*), with *r*_P_ values of .97 in both cases. All other combinations, including STAT1:STAT3 or STAT3:STAT4 (Fig. 2*B*), which readily assemble heterodimers upon their cytokine-induced tyrosine-phosphorylation (6), did not appear to heterodimerize in the absence of cytokine stimulation, and the corresponding *r*_P_ values were accordingly low (Fig. 3). Thus, unphosphorylated STATs were generally present as stable dimers, predominantly as homodimers. Heterodimers were formed only between STAT1 and STAT2 and the two very closely related STAT5 proteins. STAT6 was the only family member devoid of detectable dimerization activity.

### Latent STAT dimers require N-domain interactions

**Figure 4:**
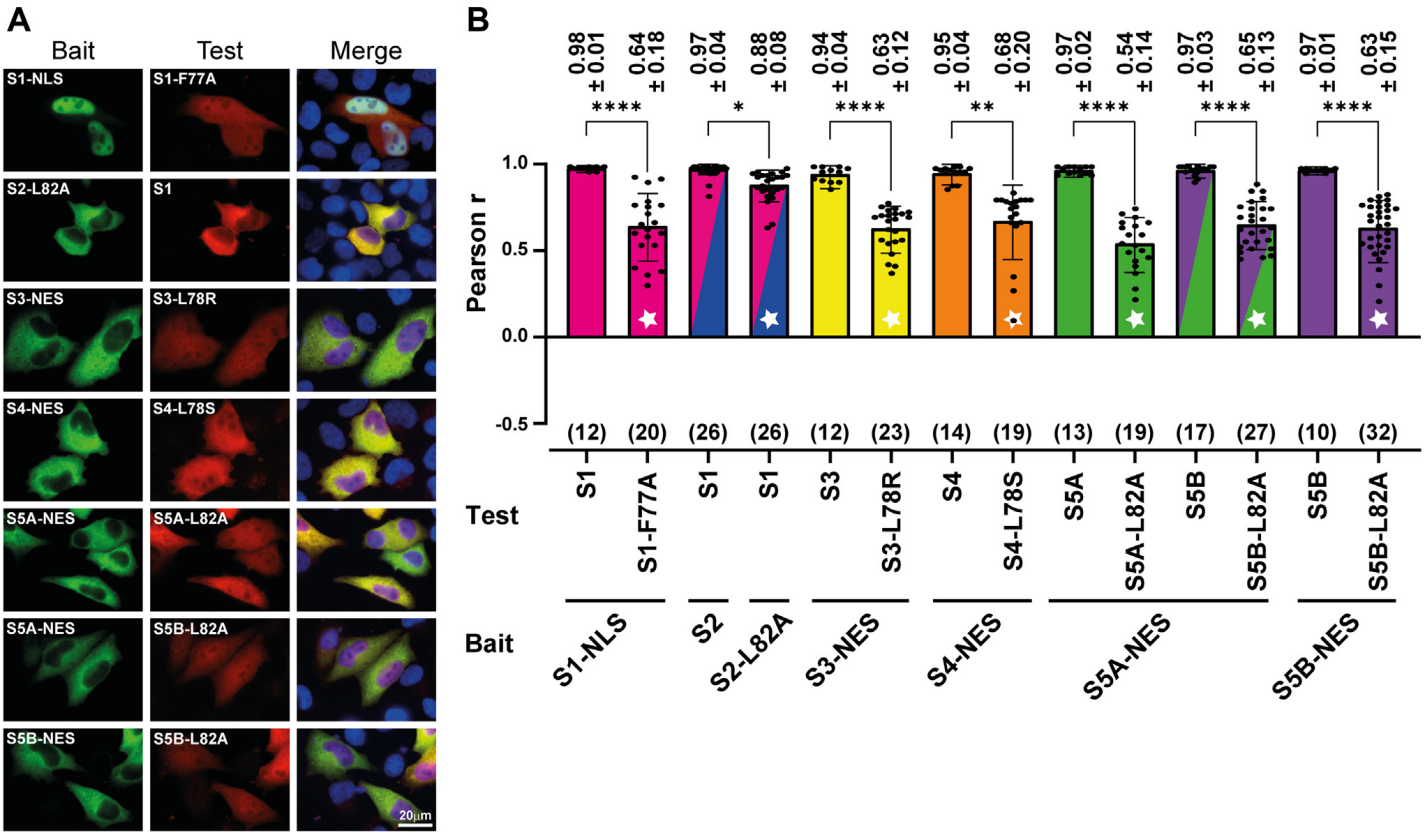
**Dimer assembly of latent STATs requires N-domain interactions.***A*, fluorescence micrographs of representative HeLa cells co-expressing bait and test STAT proteins. Bait proteins were directed to the nucleus or cytoplasm by appending heterologous NLS or NES signals (endogenous NES activity was used for STAT2), and the effect of single, potentially dimer-disrupting N-domain residues was probed on the co-localization of bait and test proteins. Presented are the *green channel*, *red channel*, and merged channels, which includes visualisation of nuclei using Hoechst dye (*blue*). *B*, bar diagram depicting corresponding Pearson correlation coefficients (r_P_) for the experiments shown in (*A*) with the individual data points (*black dots*) super imposed. Co-localization was determined as described in Figure 2*C*. ∗*p* < 0.05; ∗∗*p* < 0.01; ∗∗∗*p* < 0.001; ∗∗∗∗*p* < 0.0001 using a Kruskal–Wallis test. Rp numerical values ± SD are given above the bars; the number of cells analysed in each experiment are shown in *brackets* below bars. *White stars* signify homo- or heterodimers containing the indicated N-domain mutations. Error bars, SD.

Several lines of experimental inquiry indicate that unphosphorylated STAT dimers adopt an antiparallel conformation that is dependent on N-domain interactions (8). Deletion of the N-domain, and even single N-domain point mutations, can therefore result in the dissociation of dimers. For STAT1, N-domain residues phenylalanine 77 and leucine 78 have been shown to be critical for the assembly of unphosphorylated dimers (12), and likewise, the leucine residues 78 for STAT3 (31, 33, 34) and STAT4 (20), see also Figure 1. We, therefore, mutated the homologous N-domain residues of STAT2 (L82A), STAT4 (L78S), STAT5A (L82A), and STAT5B (L82A) to examine which of the unphosphorylated STAT dimers shared the dependence on N-domain interactions. As is shown in Figure 4*A*, all seven homo- and heterodimers were destabilized upon mutation of the same single homologous side chain in the N-domain. We concluded that the U-STAT dimers adopted similar N-domain-mediated conformations. However, the dissociating effect on the U-STAT1:STAT2 heterodimer was comparatively weak as indicated by the relatively small albeit statistically significant reduction in its *r*_P_ value (Fig. 4*B*). This could be an indication that this STAT dimer adopted an exceptionally stable conformation, which was tested next.

### U-STAT1:STAT2 heterodimers are exceptionally stable

**Figure 5:**
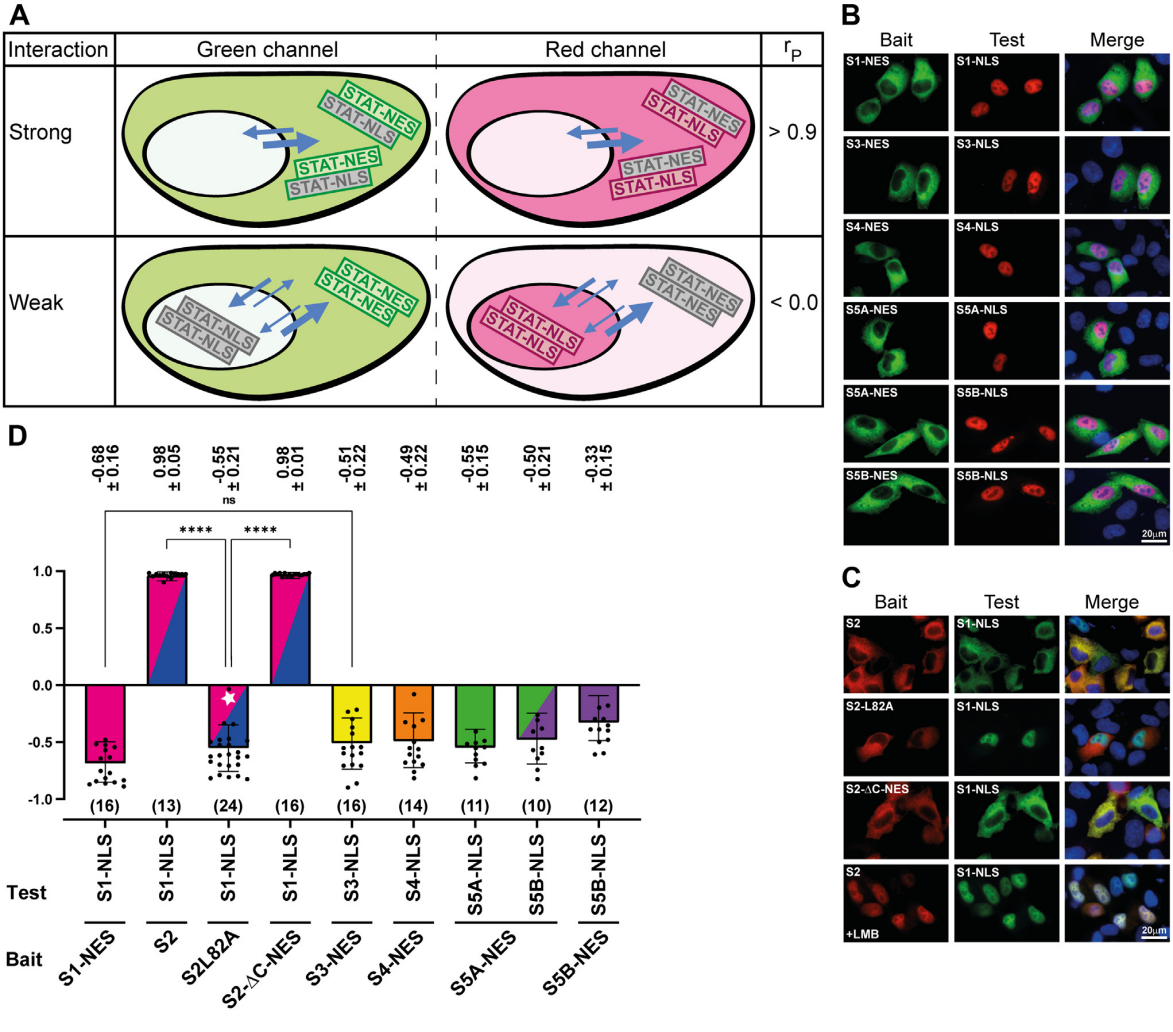
**U-STAT1:STAT2 heterodimers are exceptionally stable compared to the other latent STAT dimers.***A*, schematics describing a co-localization assay for probing the interaction strength of dimer-forming STATs. Antagonistic heterologous translocation activities (NES or NLS) are appended to bait (*green fluorescence*) and test (*red fluorescence*) proteins, respectively, whereby NES activity is dominant. *Arrow* orientation and width signify the direction and relative efficiency of signal-mediated protein translocation. *B*, fluorescence micrographs of representative HeLa cells co-expressing bait and test STAT proteins. Shown are the consequences for the co-localization of bait and test STAT proteins if they each were fused to one of the two opposed translocation signals (NES or NLS) as indicated. Presented are the *green channel*, *red channel*, and merged channels, which includes the Hoechst-stained cell nuclei (*blue*). *C*, same as (*B*). Where indicated (+LMB), cells were treated for 4 h with leptomycin B before imaging to inhibit NES-mediated nuclear export. *D*, bar diagram depicting corresponding Pearson correlation coefficients (r_P_) for experiments shown in (*B*) and (*C*) with the individual data points (*black dots*) super imposed. Co-localization was determined as described in Figure 2*C*. ∗∗∗∗*p* < 0.0001 using a Kruskal–Wallis test; ns, not significant. *White star* signifies heterodimer containing the indicated N-domain mutation. Rp numerical values ± SD are given above the bars; the number of cells analysed in each experiment are shown in *brackets* below bars. Error bars, SD.

To compare the relative binding strengths of U-STAT dimers, we co-expressed STATs that harbored opposed localization signals, namely, PKI NES or SV40 NLS. The nuclear export activity conferred by the PKI NES was determined to dominate over import activity associated with the NLS of SV40 since proteins such as GFP or GST accumulate in the cytoplasm when both signals are appended simultaneously (35). Of note, the same outcome, namely, accumulation in the cytoplasm, was observed if STAT1 was used as the acceptor of the two opposed signals (Fig. S1*A*, panel 5). We reasoned that subjecting the different dimers to these same antipodal translocation activities might reveal differences in the forces driving U-STAT association (Fig. 5*A*). As shown in Figure 5, *B* and *D*, the five U-STAT homodimers and the U-STAT5A:STAT5B heterodimer showed the same behavior, that is, the dimer subunits localized to the nucleus or the cytoplasm in accordance with their respective localization signals. We inferred that the opposed translocation forces dissociated these dimers. In notable difference, STAT2 and the nuclear-targeted STAT1-NLS variant sustained their cytoplasmic co-localization (Fig. 5, *C* and *D*), suggesting that U-STAT1:STAT2 heterodimers uniquely resisted dissociation. However, the co-localization of STAT2 and STAT1-NLS was lost upon the alanine mutation of STAT2 hot spot interface residue L82 (Fig. 5, *C* and *D*). The weakening of U-STAT1:STAT2 interactions caused by this mutation (see Fig. 4, *A* and *B*) evidently sufficed to reduce the binding affinity below the threshold required for continued co-localization, as complete separation of STAT1-NLS and STAT2 localizations was observed. To corroborate these observations, we assessed the strength of heterodimerization using C-terminally-modified STAT2 such that its less-well-characterized intrinsic NES activity was removed and replaced by the known dominant NES activity of PKI that was also used for other family members. The outcome, however, was unchanged irrespective of the specific NES activities used, as STAT1-NLS again co-localized with the STAT2-NES variant in the cytoplasm (Fig. 5, *C* and *D*). In agreement with this reasoning, the two STATs retained their co-localization, albeit in the nucleus (Fig. 5*C*), if STAT2’s CRM1-mediated nuclear export was disabled by the inhibitor leptomycin B (26). Of note, in the absence of co-expressed STAT1-NLS, treatment with leptomycin B resulted in pancellular STAT2 distribution but not its nuclear accumulation, in line with continued carrier-independent nuclear export likely being able to counter STAT2’s constitutive intrinsic import activity (Fig. S1*A*, panel 7). We concluded that the heterodimers of unphosphorylated STAT1 and STAT2 are distinguished by exceptionally strong binding interactions.

## Discussion

**Supporting Figure S3:**
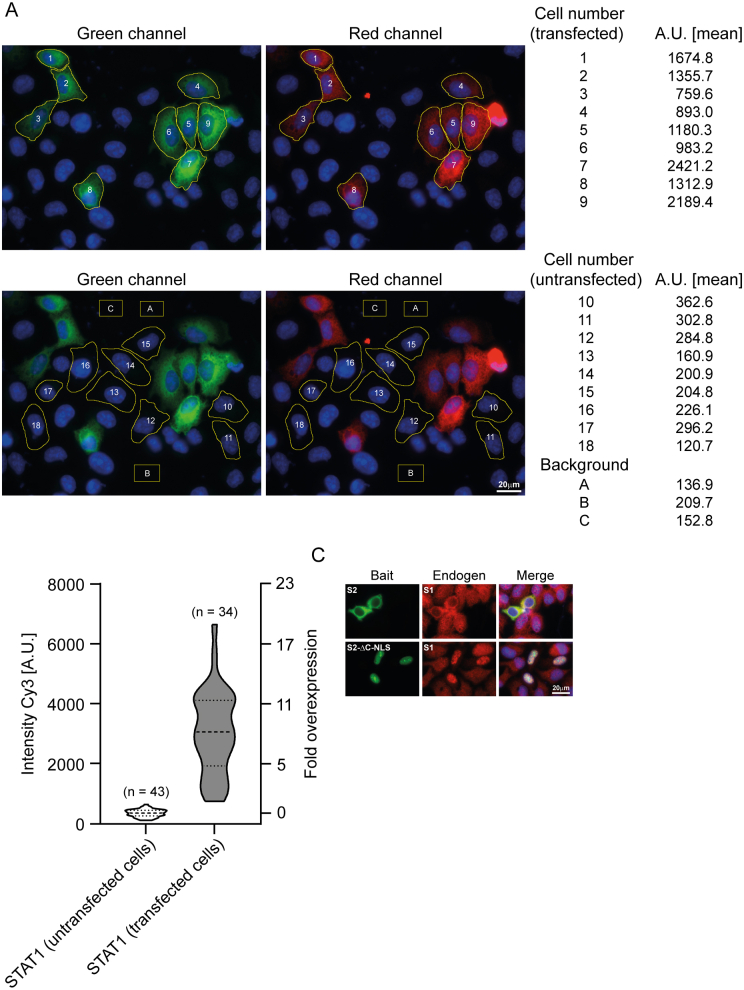
**Assessing the distribution of endogenous STAT1 and the level of STAT1 overexpression in transiently transfected HeLa cells used in this work.***A*, HeLa cells were transfected with STAT1-mEGFP expression plasmid as described under Cell Culture and Transfections in the main text using 1.2 μg DNA/well of a 24-well plate. After 20 h, cells were fixed as described under Fluorescence Imaging in the main text, followed by blocking for 1 h in 20% (v/v) FBS/PBS prior to 15 h incubation at 4 °C with primary anti-STAT1 monoclonal antibody (Becton-Dickinson 610186), washing in FBS/PBS and incubation for another hour with Cy3-coupled goat anti-mouse IgG secondary antibody (Jackson ImmunoResearch 115-165-062). Shown are four composite images of the same field of view. *Top left*, composite image of *green channel* (showing expression of transfected STAT1-mEGFP fusion protein) and *blue channel* (showing Hoechst-stained cell nuclei) with manually segmented transfected cells, based on EGFP fluorescence signals, numbered 1 to 9. *Top right*, composite of *red* and *blue* channels representing STAT1 overexpression in transfected cells. *Bottom left*, same as *top left*, but with segmentation of untransfected cells 10 to 18. Cell segmentations are based on green autofluorescence. *Bottom right*, composite of *red* and *blue channels* representing endogenous STAT1 in untransfected cells. Average red fluorescence intensities (A.U., background-subtracted) for cells 1 to 18 are listed on the *right*, together with average background fluorescence in cell-free areas recorded in the squares labelled (*A*–*C*). *B*, Violin plots summarizing the distributions of fluorescence data for untransfected and transiently transfected Hela cells, representing endogenous and overexpressed STAT1 levels. Plots include the data collected from the micrograph shown in (*A*) and four more. The *centre lines* of the plots represent the group median, the *top* and *bottom lines* represent the 75th and 25th percentiles. Fluorescence intensities are background-subtracted. The number of cells analysed are shown in *brackets*. *C*, Hela cells expressing mEGFP fusion proteins of wild-type STAT2 (S2) or NLS-tagged C-terminally truncated STAT2 (S2-ΔC-NLS) are shown after labelling with anti-STAT1 antibody to detect the distribution of endogenous STAT1 as described above. Note co-localization of the native cellular STAT1 with the transfected bait proteins. STAT1 immunostaining was done as described in (*A*) above. Fluorescence emission intensities given as arbitrary light units (A.U.).

Activated STAT proteins assemble a rather well-defined set of homo- and heterodimers that are crucial for cytokine signaling. The associations of latent STATs before activation, and how they relate to the cytokine-induced range, are less understood. Here, we report the first family-wide study on homo- and heterotypic interactions between unphosphorylated STAT proteins, which reveals that latent and activated STAT dimer repertoires overlap only partially. These results were obtained by evaluating the co-localization of nucleus- or cytoplasm-targeted STAT proteins with co-expressed wild-type or mutated family members. Like previous translocation-based methods for detecting protein–protein interactions (35, 36), our assay uses fluorophore-fused proteins and transient transfections, which can result in substantially increased protein concentrations. Because the amount of protein complexes formed is very much linked to the protein’s concentration, we compared experimental overexpression and natural STAT concentrations. STAT1 is the only family member for which the natural concentration in our HeLa cell model is known, where it is present at ∼40 nM (10). Such a low concentration is typical of transcription factors; however, STAT gene and protein expressions can be substantially increased in certain cell types, for example, NK cells contain 10- to 20-fold more STAT4 protein than non-NK cells (37). Additionally, cytokine-dependent positive feedback loops can cause STAT protein levels to rise several-fold (38, 39, 40, 41), for example, in chronic inflammatory situations such as autoimmune diseases (42), infections (43, 44, 45), or cancers (46, 47, 48). As shown in Fig. S3, STAT1 expression was increased about 3- to 15-fold in the majority of transiently transfected HeLa cells in the co-localization assay but could go up to ∼20-fold. In light of the literature, this probably includes levels at the upper limit of what can be found in nature, but not necessarily beyond it. These considerations apply to all family members, since the different STATs were expressed at similar levels in our experiments, as indicated by the recording of similar fluorescence intensities. Moreover, we assume that the endogenous STATs add to the total cellular STAT concentration, but otherwise act like the transfected counterparts and do not change their behavior. The indistinguishable binding of endogenous and transfected STAT1 to STAT2 baits is in line with this assumption (Fig. S3*C*). Although elevated protein concentrations promote self-assembly and thus may give rise to false-positive results, one STAT protein was exclusively monomeric in our hands, namely, U-STAT6. We are aware of only a single report on the complex formation of unphosphorylated STAT6. Lackmann and colleagues probed the molecular size distribution of several STAT proteins in detergent-free HeLa cell extracts and found that they resided in cytoplasmic high-molecular-weight complexes prior to any cytokine action, except for STAT6, which fractionated according to its monomeric molecular weight, consistent with our observations in living cells (49). For the other STATs, the co-localization assay indicated that they formed dimers. This is in agreement with current biochemical, structural, and *in vivo* results that demonstrate homodimerization of U-STAT1 (10, 12, 30, 50), U-STAT3 (30, 51, 52, 53, 54, 55, 56), U-STAT4 (20) and U-STAT5A (13, 57). STAT2, in contrast, did not assemble homodimers but heterodimers with STAT1. The heterodimerization of U-STAT1 and U-STAT2 has previously been documented by several independent lines of evidence (21, 22, 58, 59, 60). We observed just one additional unphosphorylated heterodimer, which was assembled by the STAT5A and STAT5B proteins, which are 93.5% identical. Of the possible further 19 heterodimer combinations, none were observed with the co-localization assay. To our knowledge, these as well as the U-STAT5 heterodimer and absence of U-STAT2 homodimers have not previously been disproven or demonstrated, with the exception of unphosphorylated STAT1:STAT3 heterodimers, which have repeatedly been reported using invasive methods such as co-immunoprecipitations (57, 61, 62, 63, 64). Several authors noted the sensitivity of U-STAT1:STAT3 interactions to salts and detergents (59, 63), they observed them only in cytoplasmic extracts (62), or at low abundance (61), suggesting weak binding forces. A quantitative assessment of U-STAT1 dimers using *in situ* single-cell pull-down of GFP-tagged STAT protein complexes reached this conclusion, too, as substantially weaker binding and rapid dissociation of U-STAT1:STAT3 compared to U-STAT1 homodimers and U-STAT1:STAT2 heterodimers was observed (60). These data and our results indicate the stability of U-STAT1:STAT3 heterodimers under certain immunoprecipitation conditions rather than constitutive complex formation within living cells.

Previous work has shown that different nuclear import and export signal sequences differ in the binding strength to their respective importin or exportin carrier molecules, whereby binding affinities measured *in vitro* and the transport activities observed in living cells generally correlate well (28, 29). Although interactions with non-carrier proteins can also have a decisive role, the balance between the strengths of import and export signals is often critical for determining the steady state localization of soluble proteins. We took advantage of this phenomenon and appended just one of the opposing signal sequences to dimer-forming STATs. Upon their co-expression, they functioned as protomers of STAT dimers, and their antagonistic translocation activities were used to assess their strength of association, which to our knowledge is a method not previously reported. Since the opposing translocation activities of PKI and SV40 are among the strongest known, it is perhaps unsurprising that they precluded co-localization indicative of dimer assembly in all but one case, namely U-STAT1:STAT2 heterodimers. Notably, the heterodimers resided in the cytoplasm, mirroring the behavior of reporters where the dominant PKI nuclear export signal and the SV40 import signal operate on the same molecule (35). This outcome suggests exceptionally strong binding interactions between the unphosphorylated STAT1 and STAT2. To date, *in vitro* binding affinities for full-length STAT2 homodimers or heterodimers with STAT1 have not been determined. However, heterodimers formed by their N-domains are of very high affinity with a dissociation constant in the low nanomolar range, thus even exceeding the affinity of full-length U-STAT1 homodimers (10, 21). Homotypic STAT2 N-domain interactions, in contrast, are several 1000-fold weaker (21), in line with the observed absence of U-STAT2 homodimers in cells.

For activated STATs, dimerization is essential to bind DNA and hence their function as transcription factors. A similar functional imperative for dimerization does not appear to exist for latent STATs. For STAT6, which was monomeric before cytokine stimulation in our assay, the recruitment to cytokine receptors, kinase interactions and tyrosine phosphorylation, and subsequent assembly of activated dimers evidently do not require latent dimers. Likewise, the events associated with cytokine-induced activation of STAT1, STAT3, and STAT5A also proceed largely undisturbed in the presence of mutations that dissociate their unphosphorylated dimers (31, 32). STAT4 is an outlier in this regard, as it requires unphosphorylated dimers to become activated and execute its cytokine-inducible activities (20). Assembly of constitutive dimers hence does not appear to be a universal requirement for the subsequent activation of STAT proteins. The expansion of the heterodimer repertoire from just two before tyrosine phosphorylation to at least eight thereafter (STAT1:STAT2, STAT1:STAT3, STAT1:STAT4, STAT2:STAT6, STAT3:STAT4, STAT3:STAT5A/B, STAT5A:STAT5B) (6) also argues against a necessity for constitutive dimers to assemble activated counterparts. Nonetheless, the binding interfaces of unphosphorylated STAT dimers share highly conserved hotspot residues that contribute substantially to homo- and heterodimerization. This suggests that there must be an enormous selection pressure to maintain these interfaces and the assembly of latent STAT dimers. In fact, mutations in STAT1 and STAT3 that dissociate latent dimers give rise to rare genetic disorders of the immune system. Of note, the alteration of a single protomer sufficed in the co-localization assay to achieve dimer dissociation, which mimics the heterozygous germline mutations in patients with STAT1 or STAT3 gain-of-function disease (65, 66). Remarkably, it is not lowered but heightened STAT activity that results from such mutations and causes disease (4). Activated STAT dimers are thought to oscillate between parallel and antiparallel conformations, whereby the antiparallel conformation is similar or identical to the conformation of unphosphorylated latent dimers (10). Importantly, antiparallel dimers of activated STATs are the substrate of tyrosine dephosphorylation (14), which is why mutations that dissociate unphosphorylated dimers can also affect activated dimers and cause resistance to dephosphorylation and heightened tyrosine phosphorylation *in vivo* (67, 68), which are defining features of STAT gain-of-function diseases. In other words, latent dimers may not be maintained as the precursors of activated STAT dimers but rather as direct products of their inactivation. Moreover, interfaces that stabilize latent dimers can participate in additional vital activities after STAT activation, such as cooperative DNA binding mediated by the N-domains, which adds to evolutionary pressures to maintain them (32, 69, 70).

In conclusion, this comprehensive analysis of latent STAT self-assembly shows that the dimerization of unphosphorylated STATs is linked to the regulation of STAT transcription activity in at least three distinct ways. One requires latent dimers for activation and (probably) inactivation and would apply to STAT4. A second way is where latent dimers seem to be dispensable for both activation and inactivation, and this applies to STAT6. The final way is where STAT activation occurs essentially normally in the absence of latent dimerization, but where the latent dimer conformation is necessary for inactivation and applies to the remaining members of the STAT protein family.

## Experimental procedures

### Cell culture and transfections

HeLa (ECACC 93021013) and HEK293T (ECACC 85120602) cells were grown in DMEM (Sigma D6429), supplemented with 10% (v/v) heat-inactivated fetal bovine serum (FBS; Sigma F9665) and 1% (v/v) penicillin/streptomycin (Sigma P0781; with 10,000 units penicillin and 10 mg streptomycin per ml in 0.9% (w/v) NaCl), in a humidified incubator with 5% CO_2_ at 37 °C. Cells were transfected at ∼80% confluence using Lipofectamine LTX according to the manufacturer’s recommendations (Invitrogen) with the following modifications. For co-localization assays, HeLa cells were transfected in 24-well plates with 1.2 μg DNA, 2 μl lipofectamine, and 2 μl PLUS reagent per well, whereby DNA encoding the bait protein was used in about 2-fold excess. Where indicated, cells were treated with 10 ng/ml leptomycin B (LMB; Cell Signaling Technology) to inactivate NES-mediated nuclear export.

### Expression constructs

STAT proteins were expressed with mCherry or mEGFP (monomeric EGFP (71)) fused to the C-terminus through cloning into pmCherry-N1 (Clontech) and pmEGFP-N1 (derived from pEGFP-N1 (Clontech), encoding the A206K mutant of EGFP), respectively. Cytoplasmic or nuclear accumulation of fusion proteins was achieved by appending canonical nuclear export (NES) or nuclear import (NLS) signal sequences. We used the NES from protein kinase A inhibitor (PKI, ^34^NSNELALKLAGLDINK^49^) and the NLS from simian virus 40 large T-antigen (SV40, ^126^PKKKRKV^132^) (28, 29). Where a single signal was appended, the respective cDNA sequence was placed between STAT and fluorophore, encoding an additional three to five heterologous residues N- and C-terminal of the NES or NLS sequence. To express STAT1 with dual NES and NLS signals, plasmid pSTAT1-NES-mEGFP was used and the SV40 NLS (plus an additional C-terminal glutamate residue) was placed downstream of the fluorophore. STAT2ΔC is a C-terminally truncated variant expressing residues 1 to 703. N-domain mutations were as follows, STAT1-F77A, STAT2-L82A, STAT3-L78R, STAT4-L78S, STAT5A-L8A, and STAT5B-L82A. Plasmid pmEGFP-mCherry encoded mEGFP fused to the N-terminus of mCherry. The two fluorophores were linked *via* an extended rigid helical linker (^1^YSDLELAEAAAKEAAAKEAAAKEAAAKEAAAKAAARDPPVAT^42^) to minimize basal Förster resonance energy transfer (72). The mutations were introduced using the Q5 site-directed mutagenesis kit (New England Biolabs). Sequences of all the plasmids were confirmed by DNA sequencing. Molecular cloning details are available upon request.

### Fluorescence imaging

Twenty hours after transfection, cells were fixed with mild agitation in 2% (v/v) paraformaldehyde in PBS for 15 min at room temperature followed by staining of nuclei for 3 min with 2.5 μg/ml Hoechst 33258 (Sigma Aldrich) and mounting in fluorescence mounting medium (Dako S3023). A Zeiss Axioplan 2 microscope with Zeiss Plan Apochromat 63× (NA = 1.4) oil immersion objective and with FITC (“green channel”) and Texas red (“red channel”) filter sets for recording EGFP and Cherry/Cy3 emissions, respectively, and Zeiss’ AxioVision 4.7 software were used for wide-field fluorescence imaging. Fourteen-bit black and white images were captured with a digital Axiocam CCD camera (Carl Zeiss Jena). Fluorescence quantitation and image analysis were performed using ImageJ (73); total cellular EGFP and Cherry signals were measured by calculating the integrated pixel intensity. All pixel values were measured below saturation limits. Adobe Illustrator (Adobe) was used to present images in the figures.

### Principle of co-localization assay and quantification of protein co-localization

**Supporting Figure S4:**
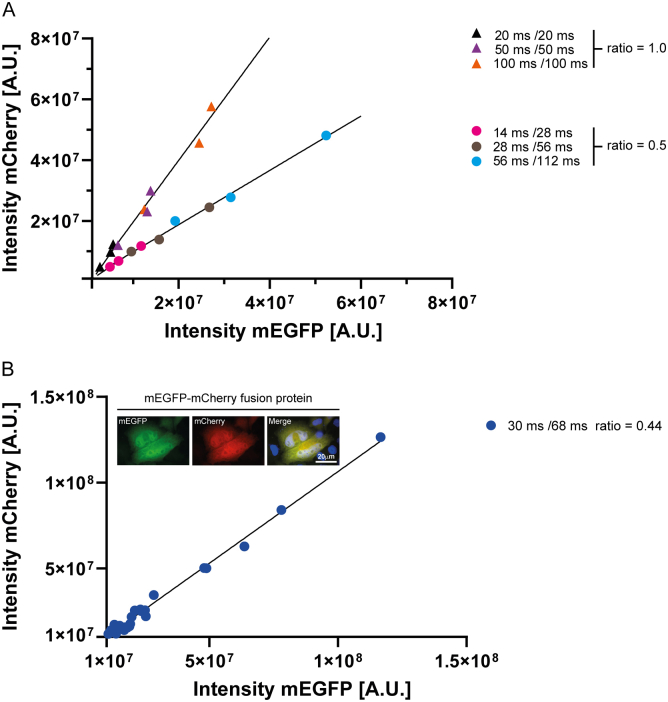
**Calibration of image acquisition settings using a mCherry-mEGFP fusion protein.***A*, Quantitative imaging results of HeLa cells transiently expressing a fusion protein consisting of mEGFP and mCherry connected by a rigid α-helical linker. At each of the two [*red*/*green*] exposure time ratios of 1 and 0.5 used, three different cells each were imaged repeatedly using the exposure times listed in the Figure. Given are the readings for individual cells and the resulting regression curves. Note linear relationships of *red* and *green* fluorescence emissions for different exposure time lengths and ratios. Fluorescence emission intensities given as arbitrary light units (A.U.). *B*, Same as (*A*), but 32 different cells were imaged using the same exposure times of 30 ms and 68 ms for *red* and *green channels*, respectively. This [*red*/*green*] exposure time ratio of 0.44 resulted in nearly identical mEGFP and mCherry fluorescence emissions over a wide range of signal intensities. It was therefore used in all experiments to quantify the relative expression levels of co-expressed STAT proteins fused to these fluorophores. In the quantitative imaging experiments shown in Figure 2, Figure 3, Figure 4, Figure 5, exposure times between 15 ms and 300 ms were used for *red* fluorescence and respective times for *green* fluorescence. The *inset* shows a representative fluorescence micrograph of HeLa cells expressing the mEGFP-mCherry fusion protein.

The assay scores the extent of co-localization of a cytoplasmic or nuclear accumulated STAT protein (STAT-NES or STAT-NLS), which functions as the bait, with another STAT, the test protein, whereby the two proteins are fused to different fluorophores. We used mCherry and mEGFP, as they are monomeric, and their emission spectra display minimal overlap (71). Co-localization is taken as a proxy for the degree of dimerization *via* homo- or heterotypic interactions. In the basic assay configuration, wild-type STAT proteins were tested. To assess dimer conformation, we tested single-point mutants homologous to key interacting residues of STAT1 (F77 or L78), which are critical for the assembly of antiparallel dimers of the unphosphorylated protein (12, 14). To assess dimer stability, bait and test proteins were furnished with opposed translocation signals (STAT-NES co-expressed with STAT-NLS) to provide forces that counteract the association of the two protomers in STAT dimers. For all assay configurations, knowledge of the relative cellular expression levels of the two co-expressed STAT proteins is necessary to ensure bait proteins are present in excess. As the STATs were expressed as fusions with mEGFP or mCherry, information about their relative expression levels could be obtained by comparison with a mEGFP–mCherry fusion protein, which expressed mEGFP and mCherry fluorophores in a known and fixed ratio of one-to-one. To facilitate quantification of the co-localization assay, all images were acquired using exposure times calibrated such that the mEGFP and mCherry fluorescence intensities were approximately equal (Fig. S4). We found that this acquisition setting reproducibly gave equivalent readings even when used on different days. Using these settings, cells were imaged, and single cells were manually segmented in ImageJ. Cells were eligible for quantitative co-localization analyses if the bait STAT protein was present from equimolar amounts to up to fourfold molar excess compared to the co-expressed test variant. To obtain quantitative estimates of the degree of co-localization in the images, Pearson (*r*_P_) and Spearman (*r*_S_, given in the source data files) correlation coefficients were calculated for individual cells with the PSC co-localization ImageJ plug-in as described (74). Both tests produce values in the range [−1, 1], 0 indicating that there is no discernible correlation and −1 and +1 meaning strong negative and positive correlations, respectively. Cells, where fewer than 1000 pixels had intensity values above that which might be considered image noise for at least one of the channels at that data point, were also deemed ineligible and excluded from further calculations. The PSC program’s default intensity setting of 40 was used as the image noise threshold. Values for Pearson correlation coefficients were calculated for 10 to 32 eligible cells per experiment and are given as means ± standard deviation (SD).

### Statistical analyses

Statistical analyses were performed in GraphPad Prism Software Version 9.3.0. D'Agostino-Pearson’s omnibus K2 was used to test for the normal and log-normal distribution of individual variables. Kruskal–Wallis test in conjunction with Dunn’s test to correct for multiple comparisons was used for hypothesis testing. The ROUT method with Q = 1% was utilized to identify outliers. Significance is designated as ∗*p* < 0.05; ∗∗*p* < 0.01; ∗∗∗*p* < 0.001; ∗∗∗∗*p* < 0.0001.

## Data availability

Quantitative fluorescence microscopy raw data from Figures 2, *A*–*C*, 3, 4, *A* and *B*, 5, *B*–*D*, and Figs. S3, *A* and *B*, and S4 are provided as publicly available source data files in the BioImage Archive (https://www.ebi.ac.uk/bioimage-archive/) under accession number S-BIAD669. All other remaining data are available within the article or supporting information.

## Supporting information

This article contains supporting information.

## Conflict of interest

The authors declare that they have no conflicts of interest with the contents of this article.
